# Effectiveness and mechanisms of mesenchymal stem cell therapy in preclinical animal models of hepatic fibrosis: a systematic review and meta-analysis

**DOI:** 10.3389/fbioe.2024.1424253

**Published:** 2024-07-22

**Authors:** Xuesong Wang, Yue Wang, Wenming Lu, Jiayang Qu, Yang Zhang, Junsong Ye

**Affiliations:** ^1^ Subcenter for Stem Cell Clinical Translation, First Affiliated Hospital of Gannan Medical University, Ganzhou, Jiangxi, China; ^2^ School of Rehabilitation Medicine Gannan Medical University, Ganzhou, Jiangxi, China; ^3^ Ganzhou Key Laboratory of Stem Cell and Regenerative Medicine, Ganzhou, Jiangxi, China; ^4^ College of Nursing, Gannan Medical University, Ganzhou, Jiangxi, China; ^5^ Rehabilitation Assessment and Treatment Center, The Third Affiliated Hospital of Zhejiang Chinese Medical University, Hangzhou, Zhejiang, China; ^6^ Key Laboratory of Prevention and Treatment of Cardiovascular and Cerebrovascular Diseases, Ministry of Education, Gannan Medical University, Ganzhou, Jiangxi, China; ^7^ Jiangxi Provincal Key Laboratory of Tissue Engineering, Gannan Medical University, Ganzhou, Jiangxi, China

**Keywords:** mesenchymal stem cell, stem cell therapy, liver fibrosis, cirrhosis, meta-analysis

## Abstract

**Background:**

Liver damage due to long-term viral infection, alcohol consumption, autoimmune decline, and other factors could lead to the gradual development of liver fibrosis. Unfortunately, until now, there has been no effective treatment for liver fibrosis. Mesenchymal stem cells, as a promising new therapy for liver fibrosis, can slow the progression of fibrosis by migrating to the site of liver injury and by altering the microenvironment of the fibrotic area.

**Aim:**

By including all relevant studies to date to comprehensively assess the efficacy of mesenchymal stem cells for the treatment of hepatic fibrosis and to explore considerations for clinical translation and therapeutic mechanisms.

**Methods:**

Data sources included PubMed, Web of Science, Embase, and Cochrane Library, and were constructed until October 2023. Data for each study outcome indicator were extracted for comprehensive analysis.

**Results:**

The overall meta-analysis showed that mesenchymal stem cells significantly improved liver function. Moreover, it inhibited the expression level of transforming growth factor-β [SMD = 4.21, 95% CI (3.02,5.40)], which in turn silenced hepatic stellate cells and significantly reduced the area of liver fibrosis [SMD = 3.61, 95% CI (1.41,5.81)].

**Conclusion:**

Several outcome indicators suggest that mesenchymal stem cells therapy is relatively reliable in the treatment of liver fibrosis. The therapeutic effect is cell dose-dependent over a range of doses, but not more effective at higher doses. Bone-marrow derived mesenchymal stem cells were more effective in treating liver fibrosis than mesenchymal stem cells from other sources.

**Systematic Review Registration:**

Identifier CRD42022354768.

## 1 Introduction

Liver fibrosis due to viral infection or alcohol intake is a major threat to human health. Persistent hepatic fibrosis is a key factor in many diseases including cirrhosis and hepatocellular carcinoma (Roehlen et al., 2020). Hepatocyte responses to inflammation allow fibrosis to develop through processes such as the formation of pro- and inflammation-suppressive cells and the recruitment of macrophages and monocytes. These inflammatory cells and chemokines accelerate the onset and progression of fibrosis by activating hepatic stellate cells which in turn accelerate fibrosis. However, there is no specific or effective treatment for fibrosis treatment (Hu et al., 2019). An increasing number of studies have shown that mesenchymal stem cells (MSCs) have been considered an attractive application in studies related to the treatment of hepatic fibrosis, with prominent characterizations of self-renewal, multidirectional differentiation, and immunomodulation. There have been demonstrated to have an ameliorative effect on the progression of hepatic fibrosis in animal models (Nomura et al., 2022; Wang et al., 2022) and clinical trials (Liu et al., 2022a).

MSCs, as one type of adult stem cells, can not only differentiate into liver cells but regulate the liver microenvironment (Eom et al., 2015). However, in clinical trials, the use of MSCs for the treatment of hepatic fibrosis has been controversial, as evidenced by the limited homing ability of MSCs after transplantation and their ability to differentiate into myofibroblasts rather than hepatocytes (di Bonzo et al., 2008). Several scholars have conducted meta-analyses of MSC therapy for liver disease, but their study subjects were mainly clinical patients. These clinical randomized controlled trial (RCT) meta-analyses targeted the recovery of overall liver function but did not involve histopathologic findings. In addition, the results of the meta-analysis are difficult to objectively reflect the effectiveness of MSCs in treating hepatic fibrosis because the physical conditions of patients were not consistent at baseline and were easily influenced by external factors (Zhou et al., 2020; Lu et al., 2023). Therefore, we conducted the first meta-analysis of MSCs therapy for hepatic fibrosis using an animal model to explore the effectiveness of MSCs in treating liver fibrosis potential precautions, and therapeutic mechanisms. In addition, this meta-analysis reports for the first time 11 key metrics of hepatic fibrosis including hyaluronic acid, laminin, hydroxyproline, collagen type III, the collagen fiber area, and transforming growth factor-β (TGF-β).

## 2 Methods

Systematic reviews and meta-analyses were interpreted and evaluated as per the Preferred Reporting Items for Systematic Review and Meta-Analyses (PRISMA) and PRISMA 2020. The registration number for this study is CRD42022354768.

### 2.1 Search strategies

**FIGURE 1:**
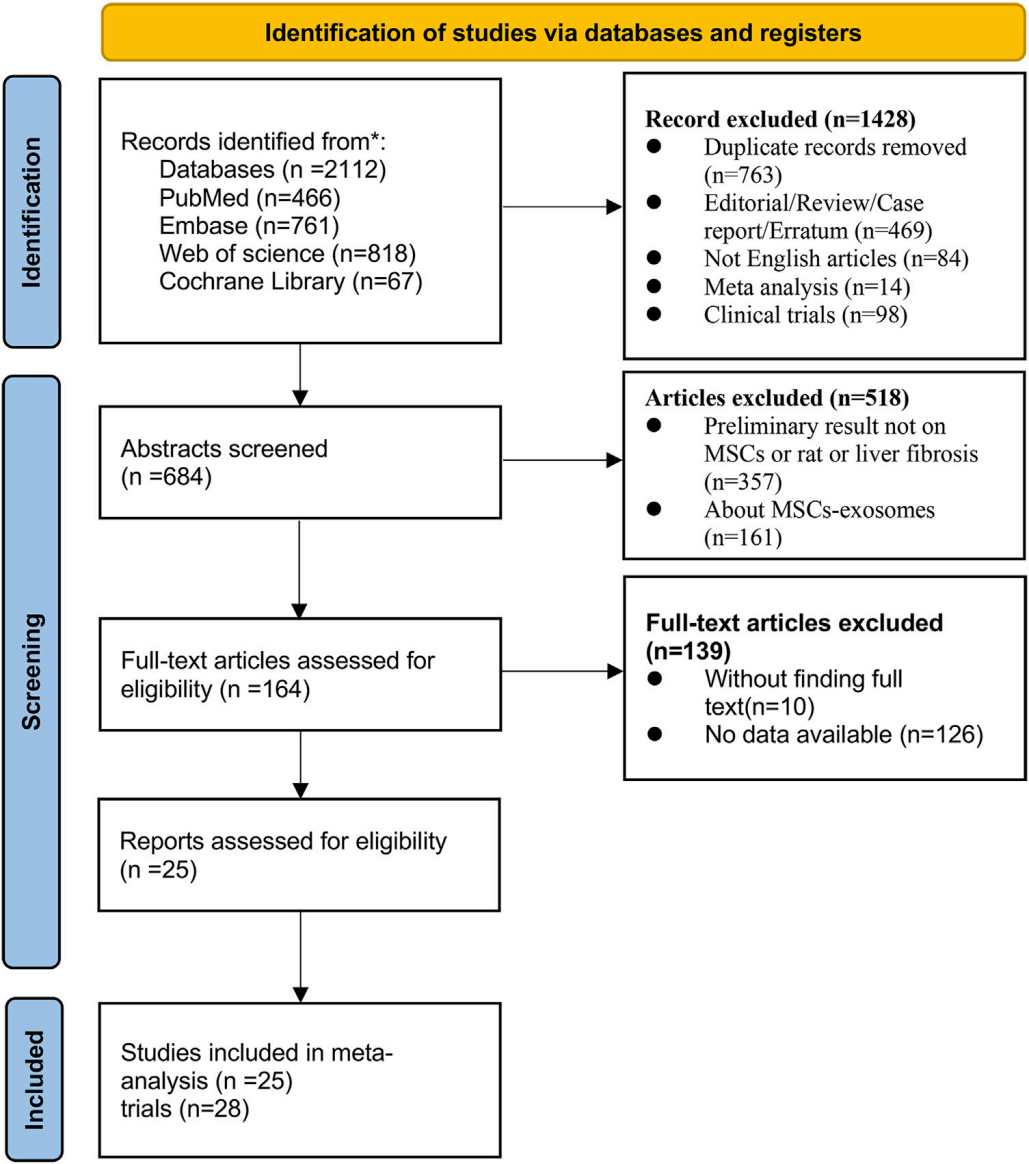
Flow diagram of the literature search and selection of studies for meta-analysis.

The first author of this article (Xuesong Wang) searched the PubMed, Embase, Web of Science, and Cochrane Library databases for the following terms (“mesenchymal stem cells” OR “ mesenchymal stromal cells” OR “mesenchymal progenitor cells” OR “wharton jelly cells”) AND (“liver fibrosis” OR “liver cirrhosis” OR “hepatic cirrhosis”) (the detailed search terms are presented in Supplementary Table S1) The database was searched from the time it was created until 2 October 2023. A total of 3 screenings were conducted, initially by screening the types of articles to exclude reviews, conferences, non-English literature, etc., followed by selecting animal model experiments that matched the topic through the titles and abstracts, and finally by reading the full text to identify a total of 25 relevant studies (28 trials) for meta-analysis (Figure 1).

### 2.2 Study selection

#### 2.2.1 Inclusion criteria

A. 1) The language used in this study was English; 2) The study involved a RCT; 3) The article involved the use of rats as an animal model of liver fibrosis.
B. In the experimental group, MSCs were used for the intervention in the rat liver fibrosis model, while in the control group, healthy rats were simply induced into liver fibrosis.
C. The study focused on the effects of MSCs on liver fibrosis and included at least one of the following metrics. 1) Albumin (ALB); 2) Alanine aminotransferase (ALT); 3) Aspartate aminotransferase (AST); 4) Alkaline phosphatase (ALP); 5) Total bilirubin (TBIL); 6) Hyaluronic acid (HA); 7) Laminin (LN); 8) Hydroxyproline (HYP); 9) Collagen fibers; 10) Type III collagen; 11) TGF-β.

#### 2.2.2 Exclusion criteria

A. The subject of the study was human, or an animal model other than rats.
B. The study is not experimental, but rather a review, conference, clinical case, or meta-analysis.
C. The complete article could not be queried or the data were incomplete, where incomplete data were included simply using statistical graphs or qualitative pictures and no quantitative data could be provided.
D. In the experimental group, MSCs were pre-treated with drugs or gene editing. This treatment may affect the results of the experiment.
E. The experiment explicitly involved extracellular vesicle therapy, not MSCs therapy.
F. The animals were not sacrificed immediately after treatment, which could have affected the outcome indicators.

### 2.3 Required data extraction

First, two researchers discussed the inclusion criteria for the data and extracted the data (Yue Wang and Wenming Lu). The extracted data were then handed over to two other researchers (Jiayang Qu and Yang Zhang), who organized and checked the data. If an article was found to have missing data or incomplete information, the corresponding author (Junsong Ye) then consulted the corresponding author of the raw article by email and ultimately supplemented the data with complete information.

### 2.4 Quality assessment

The quality of the studies was independently assessed by two researchers (Yue Wang and Yang Zhang) using an assessment tool downloaded from the Cochrane Library. The quality assessment consisted of the following steps: 1. Random sequence generation. 2. Allocation concealment. 3. Blinding of participants and personnel. 4. Blinding of outcome assessment. 5. Incomplete outcome data. 6. Selective reporting. 7. Other bias. After the assessment was performed by another researcher (Wenming Lu) for review, and when there was a disagreement, the three researchers negotiated to eliminate the disagreement and solve the problem.

### 2.5 Statistical analysis

We analyzed the mean and standard deviation of the data using software (Review Manager 5.4 and StatsSE 14) to determine the weighted mean difference (WMD) and a standardized mean difference (SMD). SMD is selected as a valid indicator when the difference in means is large. The fixed-effects model was applied on the assumption that all studies were in the same population, but animal model studies cannot be based on such a premise; therefore, a random-effects model was used. When heterogeneity was high, we also performed subgroup analyses and sensitivity analyses, which were stratified by injection route, stem cell type, stem cell injection dose, and animal model.

Funnel plots were used to assess publication bias when a single metric was greater than or equal to 10 studies. The funnel plot asymmetry was tested for accuracy by Egger’s test. Finally, the statistical significance was set at *p* < 0.05.

## 3 Results

### 3.1 Study selection

A total of 2,112 articles were identified that matched the topic of mesenchymal stem cell therapy for liver fibrosis. After removing duplicates, conference proceedings, reviews, and other studies, 25 studies (28 trials) met the inclusion criteria after the full texts of the articles were reviewed by the researchers. The complete flow chart is presented in (Figure 1).

### 3.2 Characteristics of the included studies

This study included 25 prospective studies (28 trials) involving 530 rats. A total of 278 rats with liver fibrosis were treated with MSCs. Of these 25 studies, 12 were conducted in China (Zhao et al., 2005; Chang et al., 2009; Qiao et al., 2011; Wang et al., 2012; Wang et al., 2014a; Wang et al., 2014b; Hong et al., 2014; Ma et al., 2016; Hao et al., 2017; Zhang et al., 2017; Zhang et al., 2018; Zhang et al., 2023), 8 in Egypt (Abdel Aziz et al., 2007; Ahmed et al., 2014; Raafat et al., 2015; Elberry et al., 2016; Mohamed et al., 2016; Ewida et al., 2017; Jang et al., 2018; Khalil et al., 2021), and the other 5 were from the Republic of Korea (Kim et al., 2014), Iran (Mortezaee et al., 2017), India (Aithal et al., 2018), Japan (Fathy et al., 2020), and Italy (Pietrosi et al., 2020) (Supplementary Figure S1). MSCs used to treat liver fibrosis are derived from a variety of tissues, including bone marrow, adipose tissue, the human umbilical cord, and the human amniotic membrane. Additionally, the dose and route of injection of MSCs for the treatment of liver fibrosis have been reported to vary from study to study. We extracted the basic information of each study and put all the information in Supplementary Table S2. The basic information included the authors, the year of publication, the number of rats, the type of rats, the source of MSCs, the dosage and route of MSCs injection, and the metrics corresponding to the therapeutic effect.

### 3.3 Risk assessment of bias

A total of 20 studies out of 28 trials mentioned the principle of random allocation in the text, therefore, we categorized these 20 studies as having a low risk of bias. However, the other seven studies did not mention whether they were randomized or not. No study has shown that trials were conducted by designation, concealment, or blinding of researchers. Blinding of outcome assessments was reported in only six trials and was determined to be at low risk of detection bias. Absent data, selective reporting of data, or other trial biases were not included in the meta-analysis. A total of 28 trials had reliable and acceptable methodological quality (Supplementary Figure S2).

### 3.4 Primary outcome

#### 3.4.1 ALB

**FIGURE 2:**
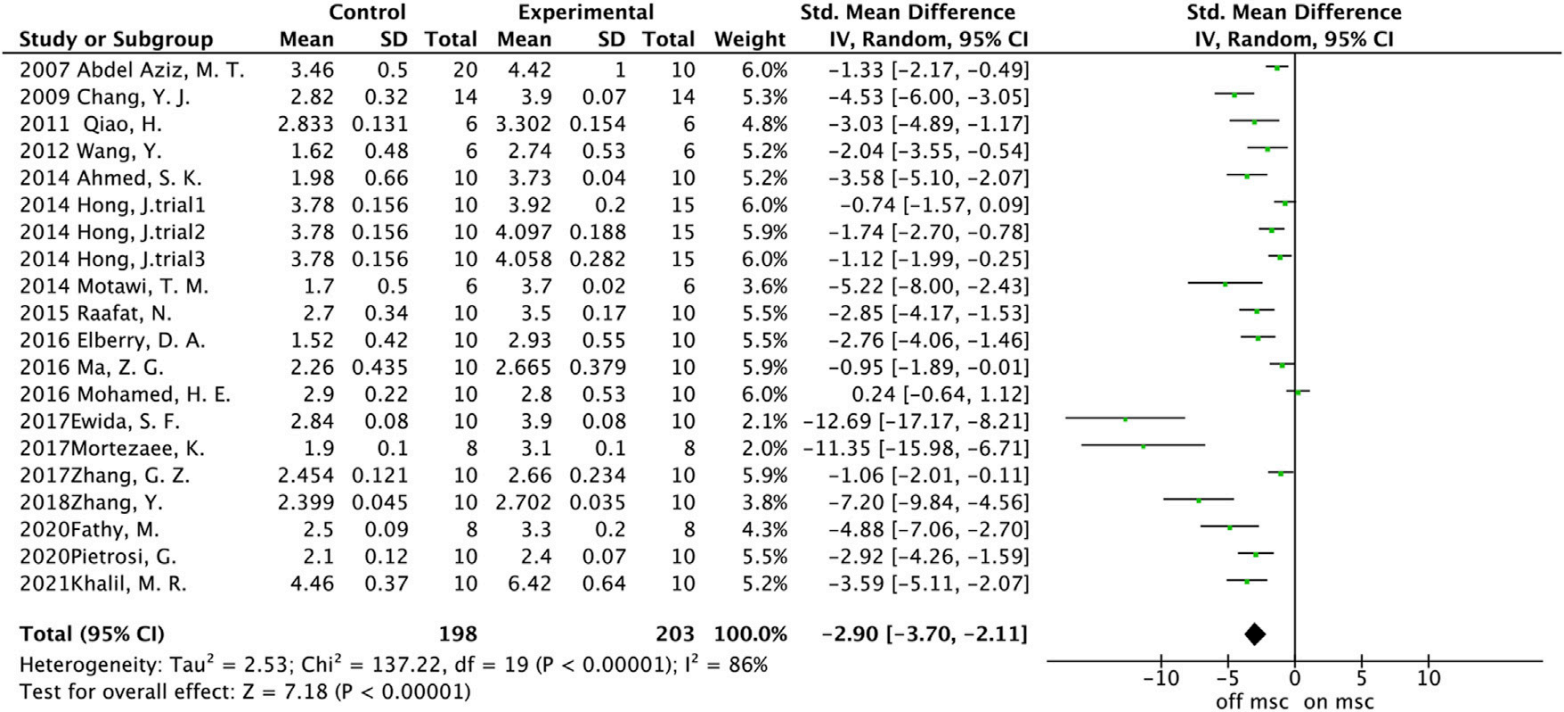
The forest plot of ALB.

**FIGURE 3:**
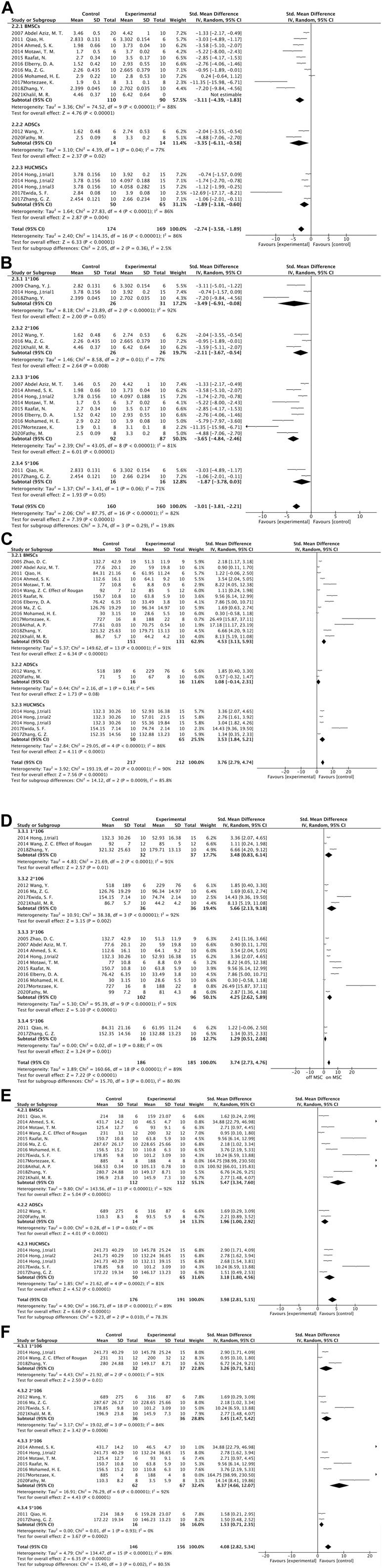
Forest plot: **(A)** MSCs source subgroup for ALB levels; **(B)** Injection dose subgroup of ALB levels; **(C)** MSCs source subgroup for ALT levels; **(D)** Injection dose subgroup for ALT levels; **(E)** MSCs source subgroup for AST levels; **(F)** Injection dose subgroup for AST levels.

**FIGURE 4:**
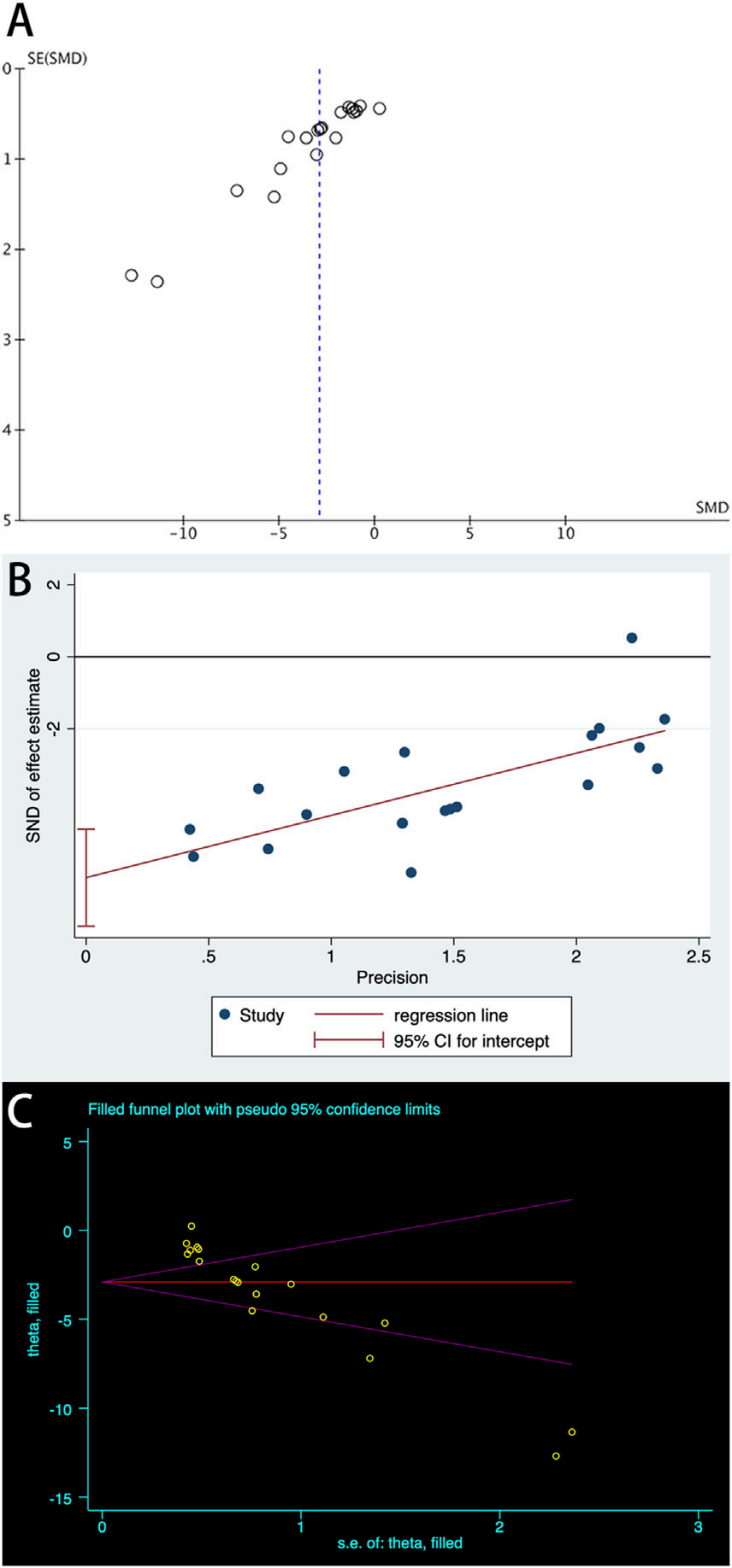
Plots of ALB: **(A)** Funnel plot with pseudo-95% confidence limits; **(B)** Egger’s publication bias plot; **(C)** Filled funnel plot with pseudo-95% confidence limits.

**FIGURE 5:**
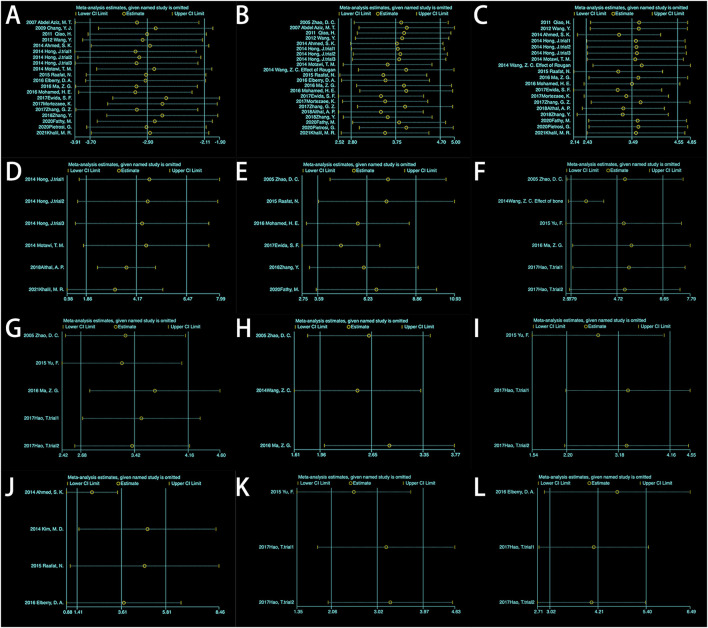
Sensitivity analysis: **(A)** Albumin (ALB); **(B)** Alanine aminotransferase (ALT); **(C)** Aspartate aminotransferase (AST); **(D)** Alkaline phosphatase (ALP); **(E)** Total bilirubin (TBIL); **(F)** Hyaluronic acid (HA); **(G)** HA (excluding 2014Wang); **(H)** Laminin (LN); **(I)** Hydroxyproline (HYP); **(J)** Area percentage of collagen fibers; **(K)** Type III collagen. **(L)** TGF-β.

A total of 20 trials from 18 studies reported differences in the serum ALB concentration between the experimental groups and control groups of MSCs for liver fibrosis (Figure 2). Although each trial showed an increase in ALB levels after MSCs treatment for liver fibrosis, there was significant heterogeneity [*p* < 0.00001, I^2^ = 86%; SMD = −2.90, 95% CI (−3.70, −2.11), *p* < 0.00001]. Subgroups were defined based on the route of injection, MSCs source, injection dose, and animal model. However, no source of heterogeneity was found (Figures 3A, B) (Supplementary Figures S3A, B). The effect of publication bias was demonstrated with a funnel plot (Figure 4A). The funnel plot showed the existence of publication bias, which was found to exist by Egger’s test (*p* = 0.000 < 0.05) (Figure 4B). The stabilization of combined effect sizes was assessed using trim-and-fill methods. A random effects model was applied (Q = 137.216, *p* = 0.000), which yielded an estimate (Est) of −2.905 and 95% CI (−3.698, −2.112) (Table 1). However, there are no virtual reports on this topic, and the results are robust and reliable (Figure 4C). Moreover, the results of the sensitivity analysis likewise confirmed the stability of the meta-analysis results, despite the presence of publication bias and high heterogeneity (Figure 5A). Therefore, it is plausible that MSCs therapy can reverse the resulting decrease in serum ALB concentration due to liver fibrosis.

**TABLE 1:** Process of the trim-and-fill method for ALB (filled meta-analysis).

Method	Pooled Est	95% CI		Asymptotic		No. of studies
		Lower	Upper	z value	*p* value	
Fixes	−1.824	−2.100	−1.549	−12.982	0.000	20
Random	−2.905	−3.698	−2.112	−7.18	0.000	

Test for heterogeneity: Q = 137.216 on 19 degrees of freedom (*p* = 0.000). Moment-based estimate of between studies variance = 2.527.

#### 3.4.2 ALT

The meta-analysis of the endpoints comprising 22 trials showed a statistically significant difference in the serum ALT between the treatment and control groups [SMD = 3.75, 95% CI (2.80, 4.70), *p* < 0.00001; heterogeneity test *p* < 0.00001, I^2^ = 89%] (Supplementary Figure S4). Subgroups were analyzed by four aspects: cell input route, source of MSCs, administration dose of MSCs, and model of animals. No significant change in heterogeneity was found (Figures 3C, D) (Supplementary Figures S3C, D). Sensitivity analyses were used to verify the stability of the results (Figure 5B), which did not significantly change the combined size effect after excluding the experiment alone. We also used funnel plots to detect publication bias (Supplementary Figure S5A). The funnel plot showed asymmetry and Egger’s test verified publication bias (*p* = 0.000 < 0.05) (Supplementary Figure S5B). The results were supplemented by the trim-and-fill approach, which uses a random effects model (Q = 198.07, *p* = 0.000) and yielded an Est of 3.747 and 95% CI (2.797,4.697). Two virtual experiments were performed (Supplementary Figure S5C), and the results were Q = 231.672, *p* = 0.000 < 0.05, and the combined effect sizes were Est = 3.395 and 95% CI (2.391,4.398) (Table 2). This finding indicates that despite publication bias, this does not affect the trend of the results, in which MSCs injections reduced ALT levels in the blood.

**TABLE 2:** Process of the trim-and-fill method for ALT (filled meta-analysis).

Method	Pooled Est	95% CI		Asymptotic		No. of studies
		Lower	Upper	z value	*p* value	
Fixes	1.924	1.643	2.205	13.421	0.000	24
Random	3.395	2.391	4.398	6.631	0.000	

Test for heterogeneity: Q = 231.672 on 23 degrees of freedom (*p* = 0.000). Moment-based estimate of between studies variance = 4.638.

#### 3.4.3 AST

Nineteen experiments reported differences in AST expression levels between the experimental and control groups. The data revealed a statistically significant difference between the two groups [SMD = 3.49, 95% CI (2.43,4.55), *p* < 0.00001; Heterogeneity testing *p* < 0.00001, I^2^ = 88%] (Supplementary Figure S6). Subgroup analyses did not reveal sources of heterogeneity (Figures 3E, F) (Supplementary Figures S3E, F). However, sensitivity analyses showed that heterogeneity did not affect the trend of the results (Figure 5C). Funnel plots were used to evaluate publication bias (Supplementary Figure S7A). The results indicated the presence of publication bias, which was likewise verified by Egger’s test (*p* = 0.000 < 0.05) (Supplementary Figure S7B). A virtual experiment was merged using the trim-and-fill method (Supplementary Figure S7C). A random effects model was used before virtual experiments were merged [Q = 149.470, *p* = 0.000, Est = 3.490, 95% CI (2.431,4.548)]. There was no effect on a verdict after combining the virtual experiments [Q = 172.332, *p* = 0.000, Est = 3.524, 95% CI (2.392,4.655)] (Table 3). These findings illustrate that the level of AST in the MSCs-treated group was lower than that in the control group.

**TABLE 3:** Process of the trim-and-fill method for AST (filled meta-analysis).

Method	Pooled Est	95% CI		Asymptotic		No. of studies
		Lower	Upper	z value	*p* value	
Fixes	2.383	2.005	2.710	14.27	0.000	20
Random	3.524	2.392	4.655	6.105	0.000	

Test for heterogeneity: Q = 172.332 on 19 degrees of freedom (*p* = 0.000). Moment-based estimate of between studies variance = 4.655.

#### 3.4.4 ALP

Six experiments containing 107 rats were involved in the analysis of differences in ALP. All six experiments showed statistically significant differences in the data between the two groups. MSCs treatment significantly reduced the expression level of ALP in rats. The data from the six experiments were then analyzed by meta-analysis using a random effects model [SMD = 4.17, 95% CI (1.86,6.47), *p* = 0.0004, Heterogeneity: *p* < 0.00001, I^2^ = 89%] (Supplementary Figure S8A). Subgroup analyses were performed using the cell injection dose, and heterogeneity was significantly reduced when the cell injection dose was 3*10^6^ [SMD = 2.92, 95% CI (1.92,3.92), *p* < 0.00001, Heterogeneity: *p* = 0.87, I^2^ = 0%] (Supplementary Figure S8B). Sensitivity analyses further tested the effect of heterogeneity on the results and presented that statistical differences between the two groups are relatively reliable (Figure 5D).

#### 3.4.5 TBIL

A total of six studies reported differences in total bilirubin expression levels in the blood of experimental and control rats. The study showed a statistically significant variation between the two groups [SMD = 6.23, 95% CI (3.59,8.86), *p* < 0.00001, Heterogeneity: *p* < 0.00001, I^2^ = 89%] (Supplementary Figure S9A). Subsequent subgroup analyses based on animal model categorization reduced heterogeneity [Wister rats: SMD = 2.65, 95% CI (1.87,3.43), *p* < 0.00001, Heterogeneity: *p* = 0.34, I^2^ = 8%; SD rats: SMD = 5.81,95% CI (3.60,8.02), *p* < 0.00001, Heterogeneity: *p* = 0.005, I^2^ = 42%] (Supplementary Figure S9B). Sensitivity analyses manifested plausible differences in total bilirubin expression levels between the two groups (Figure 5E). It indicates that MSCs effectively reduced the level of total bilirubin in the blood of rats with liver fibrosis.

#### 3.4.6 HA

Five studies containing six experiments evaluated the differences in HA expression between the two groups. A random effects model was used for the meta-analysis. The results showed that intervention of hepatic fibrosis using MSCs significantly reduced the level of HA [*p* < 0.00001, SMD = 4.72, 95% CI (2.79,6.65)]. There was a large degree of heterogeneity in the endings (*p* < 0.00001, I^2^ = 84%) (Supplementary Figure S10A). Selecting the route of injection as a means of subgroup analysis did not reveal a source of heterogeneity (Supplementary Figure S3G). However, when (Wang et al., 2014b) was excluded, it was found that the heterogeneity decreased from 86% to 0% (Supplementary Figure S10B), indicating that the study brought about heterogeneity. Sensitivity analysis using the exclusion-by-exclusion method found no effect on the results (Figure 5F), regardless of whether (Wang et al., 2014b) was excluded or not (Figure 5G), suggesting that the combined results were stable.

#### 3.4.7 LN

Three studies showed the expression levels of LN before and after MSCs treatment. A meta-analysis showed that MSCs significantly decreased the level of LN in liver fibrosis compared with control [SMD = 2.55, 95% CI (1.84,3.25), *p* < 0.00001, Heterogeneity: *p* = 0.66, I^2^ = 0%] (Supplementary Figure S11). The results showed low heterogeneity, while the outcome of the sensitivity analysis indicated that MSCs could reduce the level of LN in liver fibrosis with plausible assertion (Figure 5H).

#### 3.4.8 HYP

A meta-analysis of three trials demonstrated that there was a significant difference between the treatment group and the control group (*p* < 0.00001) (Supplementary Figure S12). The results from the fixed effect model were SMD = 3.18, 95% CI [2.20,4.16]. The results did not show the presence of heterogeneity (I^2^ = 0%). Although only three experiments yielded HYP values, the sensitivity analysis showed that the results were statistically stable when the experiments were excluded one by one (Figure 5I). The above results indicate that HYP levels can be reduced by MSC intervention in rats with liver fibrosis, and the results are stable and reliable.

#### 3.4.9 Area percentage of collagen fibers (%)

The area of collagen fibers is one of the most intuitive manifestations of hepatic fibrosis. A total of four studies reported the area of collagen fibers in results between the two groups. Comparative calculations were performed in the area between the treatment group and the control group. The results show that MSCs reduced the area of collagen fibers in the liver [SMD = 3.61, 95% CI (1.41,5.81), *p* = 0.001] (Supplementary Figure S13). However, when we performed a sensitivity analysis, we found that the results were not stable (Figure 5J). Therefore, caution is needed when extrapolating the effect of MSCs on the area of collagen fibers in hepatic fibrosis.

#### 3.4.10 Type III collagen

There were three experiments in which type III collagen was measured. The analysis was performed using a fixed-effects model, and there was a significant difference between the two groups [SMD = 3.02, 95% CI (2.06,3.97), *p* < 0.00001] (Supplementary Figure S14). Low heterogeneity was shown between the two groups (*p* = 0.39, I^2^ = 0%). To verify the reliability of the results, a sensitivity analysis was performed (Figure 5K), and the results showed that it was trustworthy that MSCs could reduce type III collagen.

#### 3.4.11 TGF-β

Two studies, including three experiments, have reported levels of TGF-ß. Fixed-effects modeling was used for the meta-analysis of TGF-β [SMD = 4.21, 95% CI (3.02,5.40), *p* < 0.00001] (Supplementary Figure S15). The outcome indicated that there was not a high degree of heterogeneity and that the difference between the two groups was stable as shown by a sensitivity analysis in which the studies were excluded one by one (Figure 5L). TGF-β is a typical effector of liver fibrosis, and MSCs reduce fibrosis by decreasing the level of TGF-β expression.

## 4 Discussion

**FIGURE 6:**
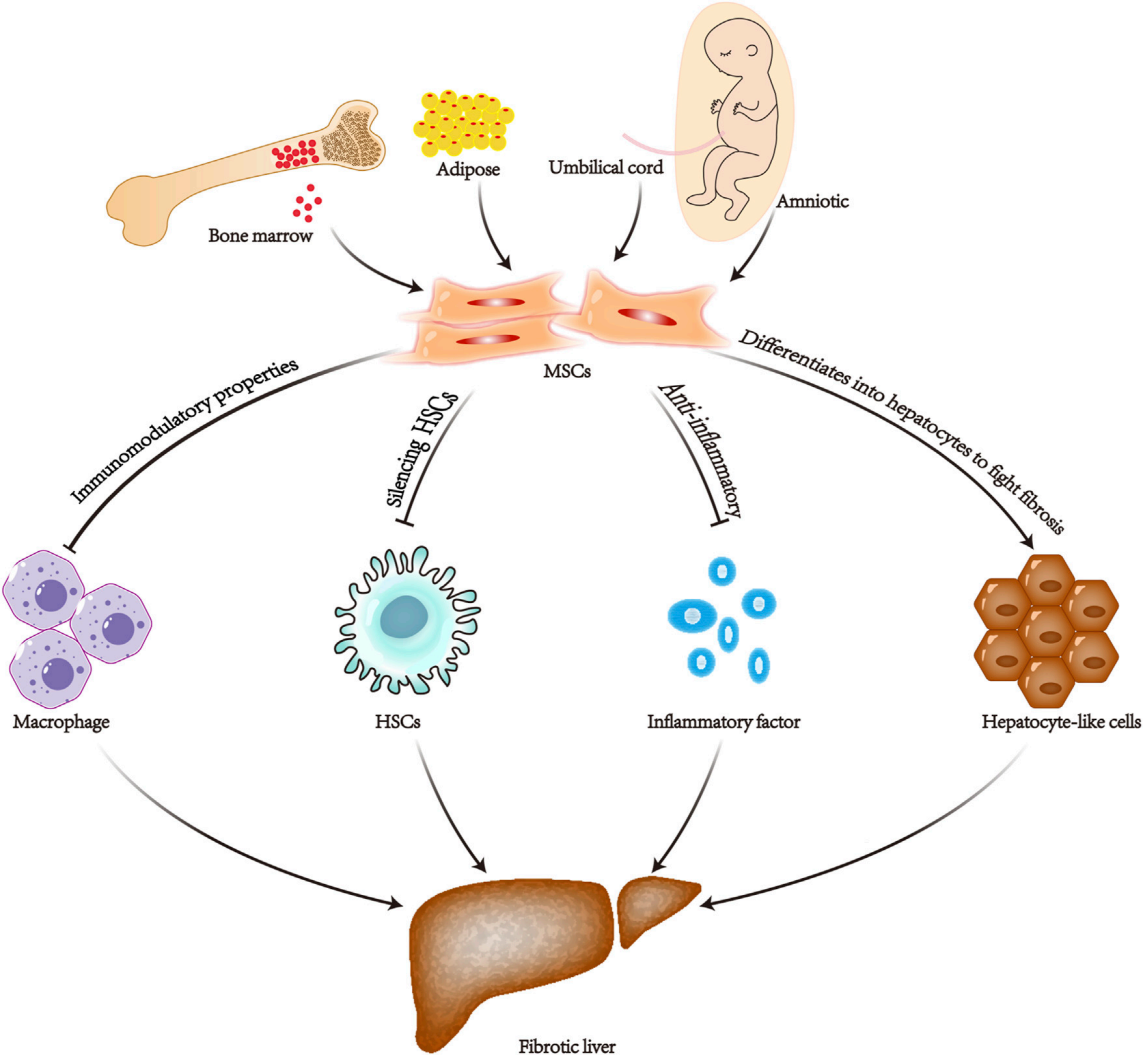
Diagram of the mechanism of mesenchymal stem cell therapy for liver fibrosis. MSCs, mesenchymal stem cells; HSCs, hepatic stellate cells.

Liver fibrosis is a crucial step in the progression of chronic liver disease to cirrhosis and even liver cancer. The pathogenesis of liver fibrosis is complicated, which makes treatment challenging. It develops mainly through the activation of myofibroblasts in the liver, which in turn secrete extracellular matrix proteins. The main source of these myofibroblasts is the resident hepatic stellate cells (Gupta et al., 2022). Hepatic stellate cells are quiescent, lipid-containing, pericyte-like cells. By tracing these resident hepatic stellate cells, 82%–96% of the total myofibroblasts were found to be involved in carbon tetrachloride-induced liver fibrosis (Mederacke et al., 2013). Therefore, it is critical to regulate the activation of hepatic stellate cells. Among the various efforts to alleviate fibrosis, mesenchymal stem cells are considered a promising therapeutic approach. It can treat liver fibrosis in various aspects. 1) Immunomodulatory properties (Xie et al., 2021; Zheng et al., 2022). 2) Differentiate into hepatocytes to fight fibrosis (Bi et al., 2017). 3) Silencing and inhibiting activation of hepatic stellate cells (Zhou et al., 2021). 4) Anti-inflammatory, antioxidant, and anti-apoptotic capabilities (Ye et al., 2019) (Figure 6). TGF-β is a major factor involved in inducing hepatic stellate cell activation (He et al., 2021).

The exploration of therapeutic mechanisms has continued in recent years. It has been found that one of the main reasons why MSCs can effectively ameliorate the level of fibrosis is due to it can significantly downregulate the mRNA expression of TGF-β and the TGF-β downstream factor SAMD3 point mRNA in myofibroblasts, as well as increase the mRNA level of SMAD7 (Yin et al., 2020; Liu et al., 2022b; Liu et al., 2022c). It has also been shown that MSCs also significantly inhibit the protein expression of SAMD2 (Kao et al., 2020). High expression of SMAD2 and SMAD3 is a major factor in the activation of the pro-fibrotic factors α-SMA and Collagen I (Peng et al., 2023). SAMD7 is an inhibitor of fibrosis and reduces hyaluronic acid levels (Su et al., 2020; Liu et al., 2022a). Bone marrow mesenchymal transplantation promotes the expression of M2-type macrophages and matrix metalloproteinase 13. Moreover, the inhibition of M1-type macrophages further inhibits the activation of hepatic stellate cells (Luo et al., 2019). Mesenchymal stem cell-based therapies have been shown to have positive effects on liver fibrosis in most animal experiments and clinical trials (Abou Rayia et al., 2023; Terai et al., 2023). However, many reports have suggested the opposite conclusion.

It has been reported that the injection of MSCs through the spleen into mice results in liver fibrosis, and only a few extraneous mesenchymal stem cells migrate to the liver (Popp et al., 2007). There are even studies claiming that bone marrow MSCs derived from adults or children by transplantation into the liver could improve α-SMA and exhibit fibrotic activity. The finding indicates potential harm to the liver parenchyma (Baertschiger et al., 2009). Moreover, some clinical trials have shown no significant improvement in liver function after MSC therapy. For example, several clinical trials have shown no significant changes in the serum ALB concentration, MELD score, or serum aminotransferase level in patients with hepatic fibrosis who underwent autologous bone marrow MSCs transplantation at the 12-month follow-up (Yang et al., 2020a). There is an urgent need to evaluate the therapeutic efficacy of MSCs in treating liver fibrosis comprehensively.

To comprehensively assess the effectiveness of MSCs in treating liver fibrosis, we performed a meta-analysis of 28 experiments using animal models. For the first time, we comprehensively pooled important indicators that can reflect the degree of hepatic fibrosis damage and performed a meta-analysis based on the pooled results. The results showed that MSCs treatment significantly ameliorated the degree of hepatic fibrosis in rats compared with the control group. Treatment resulted in similar to normal levels of ALB, ALT, ALP, AST, and TBIL for routine liver function. Heterogeneity was found to be relatively high during our analysis, and after going through subgroup and sensitivity analyses, no source of heterogeneity was found. Subsequently, we also tested for publication bias in the included studies for the three indicators of ALB, ALT, and AST, and found that publication bias did not affect the stability of the conclusions according to the trim-and-fill approach. It indicates that ALB levels can be elevated and ALT, AST, and TBIL levels can be reduced by MSCs intervention referred to liver fibrosis.

The dose of MSCs administered to patients with end-stage liver disease has usually been based solely on experience (Alfaifi et al., 2018). In 2016, scholar Suk used bone marrow mesenchymal stem cells to compare the therapeutic effects of injecting doses of 5*10^7^ once a month with 5*10^7^ twice a month for alcoholic cirrhosis patients. The results showed that there was no significant difference between the quantitative fibrosis results of the two groups (Suk et al., 2016). It has also been shown that there was a significant improvement in liver fibrosis when MSCs was injected at a dose of 1*10^7^, but not when MSCs were injected at a dose of 2*10^8^ (Amin et al., 2013; Mohamadnejad et al., 2013). Therefore, we were concerned about whether the dose of MSCs injections had an impact on the treatment effect in our meta-analysis. Interestingly in the subgroup analysis of ALB, ALT, and AST according to the MSCs injection dose, it was found that 1*10^6^ to 3*10^6^ as the number of cells increased, the greater the difference between the two groups. However, when the cell dose was increased to 5*10^6^, the therapeutic effect compared to 3*10^6^ was not satisfactory (Figures 3B, D, F). Therefore, we speculate that a limited range of MSCs doses can effectively ameliorate liver fibrosis, beyond which perhaps there is no further improvement in liver fibrosis, and even oversized doses can be harmful. Correspondingly, when the cell injection dose is within this stated range, the therapeutic effect becomes more obvious as the cell dose increases. The range of cell dosage depends on the body weight, variety of diseases, and age distribution. We are concerned about cell dosage in clinical investigations, and we will continue to follow up (Wang et al., 2023). In addition, different sources of MSCs have different therapeutic effects on liver fibrosis; however, there are no established standards for MSCs for treating liver disease, but the most well-studied MSCs in clinical practice originate from the umbilical cord and bone marrow. The umbilical cord can provide a much greater number of MSCs than the bone marrow (Mueller and Glowacki, 2001; Batsali et al., 2013). Moreover, umbilical cord MSCs have increased proliferation and differentiation capacity, lower immunogenicity, and superior allograft capacity (Cho et al., 2008; Hsieh et al., 2010). However, the results of our meta-analysis suggest that bone marrow-derived MSCs may be more effective in treating hepatic fibrosis than umbilical cord-derived or adipose-derived ones (Figures 3A, C, E). Thus, due to the advantages of umbilical cord MSCs, multiple considerations may be needed in the future before they can be used as seed cells for the treatment of liver fibrosis in the clinic.

Hyaluronic acid is synthesized mainly by hepatic stellate cells and is metabolized in the liver, and a decrease in hyaluronic acid levels in combination with TGF-β predicts a decrease in the level of hepatic stellate cell activation (Cheng et al., 2019). The results of the sensitivity analysis of the included studies indicated that MSCs attenuate hepatic fibrosis by reducing hepatic stellate cell activation.

Laminin is a non-collagenous sugar that constitutes the intercellular matrix and is synthesized in the liver mainly by endothelial cells and lipid storage cells, and together with collagen, it constitutes a component of the basement membrane (Kanninen et al., 2016). In addition, hydroxyproline, a non-essential amino acid, is one of the main components of collagen tissue and is a unique amino acid in collagen (Shi et al., 2016; Zhang et al., 2022). In clinical practice, serum laminin and hydroxyproline are mostly used as an indicator of liver disease and mainly reflect the degree of liver fibrosis activity. The results of this meta-analysis showed that the cell therapy significantly reduced the levels of laminin and hydroxyproline. The low heterogeneity of the results indicated the reliability of our analysis.

Type III collagen is generally an important index in liver biopsy and can reflect the status of collagen synthesis in the liver and is important for the diagnosis of liver fibrosis. The collagen concentration and collagen fiber area are both relatively intuitive indicators of the degree of fibrosis. It is difficult to obtain data in clinical trials and clinical meta-analyses. Our results showed that MSCs treatment effectively reduced the level of type III collagen and the area of collagen fibers in the liver. However, when sensitivity analyses were performed on experiments incorporating the fibrotic area, we found that the heterogeneity was high, and the cutoff results were unstable. Due to the insufficient sample size of the experiments, we could not specifically analyze the source of heterogeneity.

There are several limitations of the current meta-analysis that need to be mentioned. First, studies have shown that the route of cell input, the source of cells, the dose of cell input, and the animal model all affect the effectiveness of the treatment. However, due to the insufficient sample size, it is difficult to fix the other variables to further analyze which experimental protocol optimizes the therapeutic efficacy of MSCs. This also makes the heterogeneity of some indicators relatively high. Second, it would be interesting to know how safe MSCs are, especially when the dosage is different, and whether they cause adverse reactions in animals. However, during our analysis, we found that some of the studies had inconsistent numbers of models in the control and experimental groups, and the authors did not explain this inconsistency or provide the mortality rates of the animals (Abdel Aziz et al., 2007; Hong et al., 2014). Finally, we did not explain the route of injection because of the differences between animals and humans. The most common method of administration in clinical practice is peripheral intravenous injection. However, other methods of administration, such as portal vein, hepatic artery, and intrasplenic injection, need to be considered (Yang et al., 2020b). Peripheral intravenous injection has the obvious advantage of convenience; however, in the treatment of miniature pigs suffering from acute liver failure (Cao et al., 2012; Li et al., 2012), portal venous injection was found to restore liver function, whereas a similar effect was not observed for peripheral venous injection. One study compared the effectiveness of portal vein and intrasplenic injections for the treatment of liver failure and showed that portal vein injections were more effective than intrasplenic injections (Amer et al., 2011). However, given the invasive nature of portal venous injection, the choice of which route of injection should be used as a standard in the future needs to be explored in a large number of clinical randomized controlled trials.

In conclusion, our meta-analysis aggregated as comprehensively as possible the indicators concerning liver fibrosis to explore the effectiveness of MSCs in treating liver fibrosis potential precautions, and therapeutic mechanisms. The results suggest that MSCs are effective in treating liver fibrosis. Within a certain cell dose range, the higher the injection volume, the more favorable the improvement of fibrosis, but if the cell dose is blindly increased, the result will be counterproductive. Although umbilical cord MSCs are less immunogenic, bone marrow MSCs are superior in terms of therapeutic efficacy. Therefore, before MSCs can be introduced into the clinic on a large scale for the treatment of liver diseases, clinical trials are needed to determine the relevant therapeutic standards. For example, the gold standard for injection should be fixed in terms of the injection dose, injection route, and source of cells. In recent years, research related to the treatment of liver diseases by MSCs has been developing rapidly, for example, many researchers have combined gene technology and drugs to pretreat MSCs to further improve their therapeutic efficacy. There has also been much research on inducing MSCs to undergo hepatic differentiation through 2D or 3D culture *in vitro* to harvest hepatocytes for the successful treatment of liver diseases; moreover, exosomes from MSCs have also been proven to have therapeutic effects on liver diseases. However, all of the above studies may require further development before reaching the stage where they can be implemented in clinical settings.

## Data Availability

The original contributions presented in the study are included in the article/Supplementary Material, further inquiries can be directed to the corresponding author.

## Supplementary material

SUPPLEMENTARY FIGURE S1 Publication country of the included studies.

SUPPLEMENTARY FIGURE S2 Risk of bias graph and summary.

SUPPLEMENTARY FIGURE S3 Forest plot: **(A)** Route of injection subgroup of ALB levels; **(B)** Animal model subgroup of ALB levels; **(C)** Route of the injection subgroup for ALT levels; **(D)** Animal model subgroup for ALT levels; **(E)** Route of injection subgroup for AST levels; **(F)** Animal model subgroup for AST levels; **(G)** Injection dose subgroup for HA levels.

SUPPLEMENTARY FIGURE S4 The forest plot of ALT.

SUPPLEMENTARY FIGURE S5 Plots of ALT: **(A)** Funnel plot with pseudo-95% confidence limits; **(B)** Egger’s publication bias plot; **(C)** Filled funnel plot with pseudo-95% confidence limits.

SUPPLEMENTARY FIGURE S6 The forest plot of AST.

SUPPLEMENTARY FIGURE S7 Plots of AST: **(A)** Funnel plot with pseudo-95% confidence limits; **(B)** Egger’s publication bias plot; **(C)** Filled funnel plot with pseudo-95% confidence limits.

SUPPLEMENTARY FIGURE S8 The forest plots of ALP. **(A)** ALP levels; **(B)** The cell injection dose subgroup of ALP levels.

SUPPLEMENTARY FIGURE S9 The forest plots of TBIL. **(A)** ALP levels; **(B)** Animal model subgroup of TBIL levels.

SUPPLEMENTARY FIGURE S10 The forest plots of HA. **(A)** HA levels; **(B)** HA levels when 2014 Wang was excluded.

SUPPLEMENTARY FIGURE S11 The forest plot of LN.

SUPPLEMENTARY FIGURE S12 The forest plot of HYP.

SUPPLEMENTARY FIGURE S13 The forest plot of the area percentage of collagen fibers.

SUPPLEMENTARY FIGURE S14 The forest plot of type III collagen.

SUPPLEMENTARY FIGURE S15 The forest plot of TGF-β.

## Abbreviations

MSCs, Mesenchymal stem cells; BMSCs, Bone marrow MSCs; HUCMSCs, Human umbilical cord MSCs; ALB, Albumin; ALT, Alanine aminotransferase; ALP, Alkaline phosphatase; HA, Hyaluronic acid; HYP, Hydroxyproline; CCl4, Carbon tetrachloride; DMN, Dimethylnitrosamine; HSCs, Hepatic stellate cells; ADSCs, Adipose MSCs; HAMSCs, Human amniotic MSCs; AST, Aspartate aminotransferase; TBIL, Total bilirubin; LN, Laminin; TGF-β, Transforming growth factor-β; DEN, Diethylnitrosamine; TAA, Thioacetamide.
