# Supplementary material for: Effectiveness and mechanisms of mesenchymal stem cell therapy in preclinical animal models of hepatic fibrosis: a systematic review and meta-analysis
Source: Front Bioeng Biotechnol. 2024 Jul 22;12:1424253. doi: 10.3389/fbioe.2024.1424253 (PMC11299041; doi:10.3389/fbioe.2024.1424253)
Supplement: Supplementary file 12 [file Table2.DOCX]

**Characteristics of the included studies**

**Table1**

Wang, Z. C. A^[17]^; Wang, Z. C. B^[18]^; CCl4,carbon tetrachloride; DEN, diethylnitrosamine; DMN, dimethylnitrosamine; TAA, thioacetamide; BDL, bile duct ligation.
